# Effect of Ingestion Exposure of Selected Insecticides on *Coccinella septempunctata* and *Harmonia axyridis* (Coleoptera: Coccinellidae)

**DOI:** 10.3390/insects12050434

**Published:** 2021-05-11

**Authors:** Jacek Piotr Twardowski, Michał Hurej, Kamila Twardowska

**Affiliations:** Department of Plant Protection, Wroclaw University of Environmental and Life Sciences, Grunwaldzki Sq. 24a, 50-363 Wroclaw, Poland; michal.hurej@upwr.edu.pl (M.H.); kamila.twardowska@upwr.edu.pl (K.T.)

**Keywords:** ladybirds, coccinellidae, aphicides, toxicity, mortality, survival

## Abstract

**Simple Summary:**

In chemical plant protection against pests it is very important to know the indirect effects of insecticides on non-target organisms. This research shows the indirect effects of two insecticides (thiacloprid and lambda-cyhalotrin as active substances) on two different species of ladybirds, i.e., native in Europe *Coccinella septempunctata* and alien invasive species *Harmonia axyridis*, which were fed with aphids previously intoxicated by an insecticide. The products tested killed most instars of both ladybird species within 3 h of the start of the ingestion of the intoxicated prey. The action of thiacloprid, to which the predators are exposed by the same route, was more extended in time in both the coccinellids, and more variable between their developmental instars. The applications of thiacloprid caused different responses in the two predator species, which is nevertheless variable across instars.

**Abstract:**

The sensitivity to thiacloprid and lambda-cyhalothrin ingested from prey organisms was studied in *Coccinella septempunctata* and *Harmonia axyridis*, since the effect of ingestion exposure to these insecticides is unknown in these species. All developmental stages of the ladybirds were fed on *Acyrthosiphon pisum* treated with half or full field rate of the insecticides. Almost all instars were killed within 3 h of the start of ingestion of lambda-cyhalotrin-treated prey. The action of thiacloprid was more extended in time in both coccinellids and more variable between their instars. Reducing the field rate of lambda-cyhalothrin seems to have no practical value for the survival of either of the coccinellid species. Contrastingly, using half instead of the full field rate of thiacloprid may enhance the chances of survival in L1, L2, and L4 larvae of both species. Of all developmental stages tested, the survival dynamics of the adults of either species are closest to one another, whereas the apparent difference in the species response to the dose rate of thiacloprid was found in the L4 stage.

## 1. Introduction

The seven-spot ladybird, *Coccinella septempunctata* Linnaeus, 1758, is an abundant species in a wide range of Eurasian, African, and North American agroecosystems [1]. European populations of this coccinellid have been intensively studied [2,3,4], as it preys upon several economically important aphid species. Until now, at least 27 aphid species have been recorded as suitable food for *C. septempunctata* [3,5]. It has the status of native species in Europe and is often listed as a natural enemy of many insect pests in variety of crops, such as alfalfa, cereals, clover, maize, faba bean, oilseed rape, pea, potato, and sugar beet [6,7,8]. Because it is also common in orchards, on wild herbaceous plants, shrubs, and trees, *C. septempunctata* is considered the most abundant of all the coccinellid species in Poland [9].

The susceptibility of *C. septempunctata* to some pesticides was studied by Bozsik [10]. This author lab-tested five different insecticides at their field rates, for their acute detrimental side-effects on adult seven-spot ladybirds. Biological product based on *Bacillus thuringensis*, proved safe for the population of adults, was also used in his work. Pyriproxifen, deltamethrin + heptenophos, and lambda-cyhalothrin were found to be moderately harmful to adults of *Coccinella septempunctata.* More recent studies were conducted by Sattar et al. [11]. Twelve active substances were analyzed, including lambda-cyhalothrin. It was observed that this pyrethroid reduced the locomotion in *C. septempuctata*. The efficacy of lambda-cyhalothrin on the coccinellid population may demonstrate that the effect of insecticide pressure and their susceptibility to certain insecticides are similar across the world. New research on the effects of various insecticides on ladybirds has been carried out by many authors, including Skouras et al. [12,13], Tengfei et al. [14], and Zhixin et al. [15].

In contrast to *C. septempunctata*, the harlequin ladybird, *Harmonia axyridis* Pallas, 1773, the species native to Asia, is considered an invasive, alien ladybird in Europe, to where it had been introduced as biological control agent against aphids and coccids [16,17]. Nevertheless, the expansion of the species over many other parts of the world was mainly spontaneous. Adults of the species are known to have strong dispersal capacity [18,19] and studies in North America have shown that *H. axyridis* can quickly colonize large areas [20]. The predator has been spreading rapidly in Europe, particularly since 2002, and populations of the species now exist in at least 26 European countries [21]. The first population of *H. axyridis* in Poland was recorded in 2006, but it has now colonized the entire country [22,23]. It occupies almost the same agricultural, arboreal, natural, and semi-natural habitats as the native *C. septempunctata*.

*H. axyridis* is known for its strong impact on native ladybird species [24]. Compared to other aphidophagous coccinellid species, the harlequin ladybird has been shown to have superior competitive abilities regarding its feeding rate [20], intraguild predation [25,26,27], and interactions with natural enemies [28,29,30]. Another factor possibly responsible for the predominance of *H. axyridis* in various coccinellid assemblages could be its different susceptibility to at least some of the currently used insecticides, as compared to other, native species. The direct impact of pesticides on *H. axyridis* has been widely studied under field and laboratory conditions, and for application to different agricultural systems [18,31,32,33,34,35,36,37], showing that the species’ susceptibility to different pesticides is variable and depending on the developmental stage. Some studies show that adults are more often less susceptible than are immature instars [18,38]. Coccinellids may be also exposed to insecticides indirectly, by consuming the insecticide-treated prey [39,40].

Each pesticide product should be studied not only to measure direct acute toxicity to beneficial arthropods but also towards different sublethal effects inducing changes in their physiology, development, adult longevity, fecundity, consumption rate, or any other effects. Adverse impacts of insecticides on beneficial insects should be avoided by choosing an appropriate active ingredient and dose rate. An extensive literature review on this topic was accomplished by Desneux et al. [41]. Since that time several new studies have been performed on this issue [28,42,43,44,45]. Likewise, comprehensive studies on the side effects of even those commonly used insecticides are required to limit the adverse effects on adults and larvae of beneficial arthropods, including ladybirds.

The present paper reports the ingestion exposure of two insecticides commonly used against aphids and other pests in Europe, to all larval instars and adults of *C. septempunctata* and *H. axyridis*, when fed on the insecticide-treated prey. The working hypothesis of the present study is that the two predators vary in their susceptibility to aphicides applied to control their prey.

## 2. Materials and Methods

### 2.1. Insects

The study was carried out as a series of laboratory tests. The ladybird beetles were sampled from the field and reared in the laboratory. Adults of *C. septempunctata* were collected from winter wheat and the adults of *H. axyridis* were sampled from *Prunus domestica* subsp. *syriaca* (Syrian plum), in summer 2017. The number of beetles obtained was at least 300 for each species to receive a uniform material for later testing. Ladybird beetles were reared at 25 ± 1 °C, 75 ± 5% RH, and 16L:8D photoperiod, in the net cages of 50 × 50 × 50 cm size, on pea seedlings (cv. Cysterski) grown in pots in a growth chamber and infested by the pea aphid, *Acyrthosiphon pisum* (Harris). The pea seedlings were grown in separate cages. One week old plants were infested by gently shaking a pot of aphid-infested plants to dislodge pea aphids onto new seedlings. The aphids’ population built-up on newly infested plants for 10 days and were then transferred to beetle-rearing cages twice a week. The eggs of the coccinellids collected daily were monitored for eclosion. Newly hatched larvae were placed individually into plastic Petri dishes of the ø 90 mm and 10 mm height, and provided daily with *ad libitum* supply of *A. pisum* with 10% sucrose water. They were monitored daily to record the number of molts until they reached the stage appropriate for the test.

### 2.2. Insecticide Preparation

Commercially available insecticides Karate Zeon 050 CS (0.050 kg/L lambda-cyhalothrin, Syngenta, further abbreviated as Karate) and Calypso 480 SC (0.480 kg/L thiacloprid, Bayer, further abbreviated as Calypso) were used in the tests. Lambda-cyhalothrin and thiacloprid are group 3A (pyrethroids) and group 4A (neonicotinoids) insecticides respectively, according to IRAC classification [46]. Lambda-cyhalothrin is an agonist of the sodium channel, and thiacloprid—an agonist of the nicotinic acetylcholine receptor (NAChR) in the nerve cells. Suspensions of the insecticides in distilled water were prepared for each test at concentration equivalent to 100% or 50% of the recommended field rate of the product diluted in 300 L water, as recommended on the labels for the control of aphids in the crops most commonly grown in Poland: 16.7 mg/L or 8.3 mg/L lambda-cyhalothrin and 256.0 mg/L or 125.0 mg/L thiacloprid respectively. The suspensions were prepared no sooner than 15 min before the start of each test.

### 2.3. Tests

*Acyrthosiphon pisum* was reared on pea seedlings as previously described. The aphid-bearing stems and leaves of pea were excised from the whole plant, dipped in the insecticide suspension or in distilled water (reference, i.e., untreated control) for 5 s, and then dried in the fume hood for 20 min. Thus, intoxicated prey were then dislodged onto Petri dishes using a fine brush, and stored there until they were offered to ladybirds, usually for no longer than 20 min. This procedure made it possible to have good insecticide coverage of aphids and to avoid direct contact of predators with the treated plants. Insecticide-treated or water-treated aphids were added to each Petri dish containing 5 individuals of the same instar of *C. septempunctata*, or *H. axyridis*, similarly as described by Hurej and Dutcher [39]. The adults and the L1–L4 instars of the predators were taken for each test the time they hatched or moulted in a suitable number. Starved adults were used in the tests as 4-day old individuals. Mortality was recorded at 1, 3, 24 and 48 h after the first prey was offered. Predatory larvae and adults were considered dead if they did not move their legs when stimulated with a fine brush. In each run of the experiment 5 replicates were used in the treatment, and another 5 replicates used in the respective reference test carried out on the same day. Each replicate used 5 coccinellid individuals of the same instar.

### 2.4. Statistical Analysis

Mortality was expressed in percentage values. Mortality correction, based on survival rates from the relevant reference tests, was calculated according to Abbott [47]. The raw data did not show normal distribution and the parametric ANOVA was not carried out. Instead, Kaplan-Meier (K-M) survival analysis and the Mantel-Haenszel test were used to plot survival functions for each predator species and to find out differences between them. Both procedures are available in Statistica 13 software (1984–2016 Dell Inc., Round Rock, TX, USA).

## 3. Results

### 3.1. Survival; Lambda-Cyhalothrin

**Figure 1:**
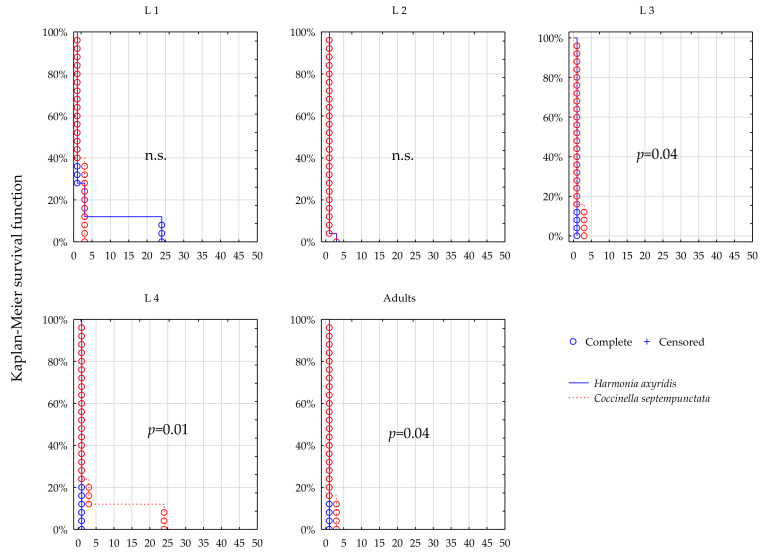
Survival dynamics of larvae and adults of the two predators after foraging on the prey treated with Karate Zeon at 100% recommended field rate.

**Figure 2:**
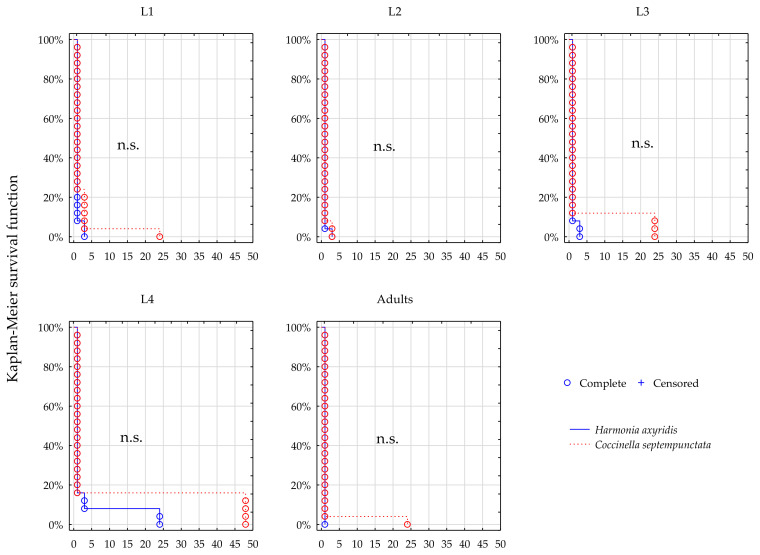
Survival dynamics of larvae and adults of the two predators after foraging on the prey treated with Karate Zeon at 50% recommended field rate.

**Figure 3:**
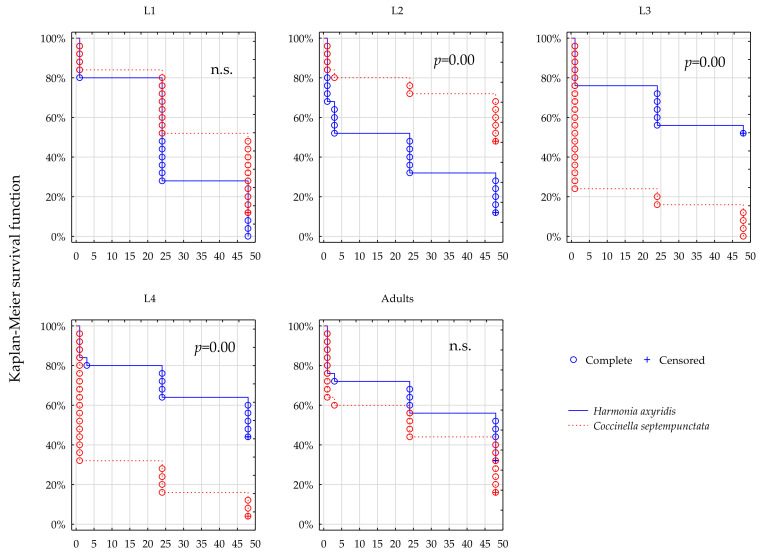
Survival dynamics of larvae and adults of the two predators after foraging on the prey treated with Calypso at 100% recommended field rate.

**Figure 4:**
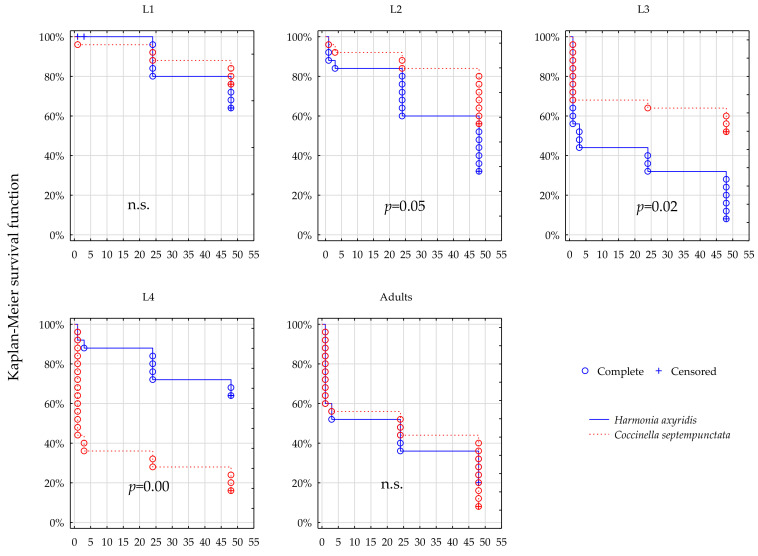
Survival dynamics of larvae and adults of the two predators after foraging on the prey treated with Calypso at 50% recommended field rate.

Comparing survival function for *H. axyridis* and *C. septempunctata* (Figure 1, Figure 2, Figure 3 and Figure 4, Table 1) suggests that survival dynamics of the two predators were more often different from one another after their prey was exposed to thiacloprid (Calypso), compared to when they were exposed to lambda-cyhalothrin (Karate Zeon).

At the full field rate of Karate, significant differences were found between the two species in survival of L3, L4, and adults, and in these instars the longer survival of *C. septempunctata* was observed (*p* = 0.04, *p* = 0.01, *p* = 0.04, L3, L4, adults respectively, Figure 1). Survival of the L1 and L2 instar seems similar in both species (n.s., Figure 1). At the half field rate of Karate the survival dynamics of the two predators were statistically uniform (n.s., Figure 2).

### 3.2. Survival; Thiacloprid

After exposure to the prey treated with Calypso, survival dynamics varied significantly between *C. septempunctata* and *H. axyridis* in the instars L2–L4, at both field rates of the product (Figure 3 and Figure 4). The survival of L2 was apparently higher in *C. septempunctata* compared to *H. axyridis* (Figure 3 and Figure 4 L2, Table 1, Calypso 100% and 50% field rate, L2: *p* = 0.005 and *p* = 0.051 respectively). The L4 plots demonstrate large difference between survival of the two predators at both field rates of Calypso, but contrary to L2 the survival of L4 instars was higher in *H. axyridis* than in *C. septempunctata*, irrespective of the dose rate (Figure 3 and Figure 4 L2, Table 1, Calypso 100% and 50% field rate, L4: *p* = 0.005 and *p* = 0.000 respectively). The survival of L3 instars was significantly different between the predators (Figure 3 and Figure 4 L3, Table 1, Calypso 100% and 50% field rate, L4: *p* = 0.005 and *p* = 0.016 respectively). Survival of the L1 and of the adults compared between the two species at the same field rates are similar and statistically uniform.

## 4. Discussion

Insecticides are chemical compounds commonly used to control arthropods pests. However, at the same time they may have negative impacts on non-target beneficial organisms, important as natural enemies [40,45]. Both acute and sublethal effects have often been studied on different organisms, including ladybirds [36,37,38,42,45,48].

The two insecticides used in the present study show different modes of action. Accordingly, apart from the inter-specific variation in mortality, differences were demonstrated between the two products in their activity on the same predator species. Lambda-cyhalothrin was shown to act quickly in all instars of both predators and, in spite of the indirect exposure, it left almost no survivors after 3 h when applied at the full dose rate, or after 24 h, when used at half the field rate.

Significant variations in susceptibility to lambda-cyhalothrin were observed in relation to different coccinellid species occurring within cotton fields in Brazil [49]. In this study, seven and eighteen populations of lady beetles exhibited greater values of LD_50_ and LD_90_, respectively, than the highest recommended field rate of lambda-cyhalothrin (20 g a.i./hectare ≈ 0.2 g a.i./L). In another study, Jalali et al. [50] demonstrated that lambda-cyhalothrin, while consumed with the treated aphids, was 25 times as toxic to adults and 8 times as toxic to larvae of *A. bipunctata*, as was dimethoate, the organophosphate synaptic poison of acetylcholinesterase. While comparing the toxicity of different insecticides to two aphid species and to *H. axyridis*, Cho et al. [51] found alphamethrin “much safer to the predator than to the pest”. Yet the esfenvalerate and the alphamethrin (=alpha-cypermethrin, CAS No.: 67375-30-8), similarly to the lambda-cyhalothrin used in the present study, all belong to the so called “type II pyrethroids”, i.e., the compounds containing the ∝-cyano group [52]. Despite their chemical relatedness, their toxicity is varied in different insect species and their instars. Our results indicate that *C. septempunctata* and *H. axyridis* are two other species next to *A. bipunctata* which are susceptible to lambda-cyhalothrin when feeding on contaminated aphids.

The insecticidal activity of thiacloprid was, in the present study, more extended in time but more variable between developmental stages of each species. It allowed a considerable number of individuals to survive beyond the 48 h limit of the test, as mirrored in the censored observations, and, contrary to lambda-cyhalothrin, the mortality brought about by thiacloprid was, most often, in L3 and L4 instars, lower in *H. axyridis* when compared to *C. septempunctata*. However, it was the L2 instar of *C. septempunctata* that survived thiacloprid exposure significantly better than *H. axyridis*.

What should be considered unusual is the reversed dose/mortality response observed in *H. axyridis* after indirect exposure to thiacloprid. It seems plausible that, contrary to Karate, Calypso used at concentrations equivalent to 100% of the field rate exerts some kind of deterrent effect on predators (whether via olfactory or gustatory route), making it periodically restrain from the consumption of prey and subsequently resume foraging, the phenomenon taking place repeatedly. This might have reduced the overall toxin consumption beyond the level achieved in 50% field rate treatment, simultaneously allowing an extra time interval for its degradation in the insect organism and resulting in lower mortality. It is also known that the voracity of adults and the oldest larvae is greater than that of the younger instars in most ladybird species. It seems possible that the lower content of toxin in the prey exposed to 50% of the field rate is compensated for by the higher consumption rate of it and that, conversely, at the 100% field rate, a similar amount of toxin is taken up at a more rapid pace, as its content in the prey tissue is higher. Consequently, although the mortality induced by the prey exposed to the higher field rate ensues at an earlier time than that brought about by the lower field rate, the two survival dynamics observed at both field rates closely follow one another for most of the time.

The response to thiacloprid of the tested predators observed in the present study may be described as, most of the time, less predictable and more variable between adults and larvae (in both species), contrary to their rapid and rather uniform response to lambda-cyhalothrin. The results presented indicate that, contrary to lambda-cyhalothrin, using thiacloprid may sometimes work in favor of *H. axyridis* (based on the higher survival of L3 and L4 instars), but the data for L2 instar testify to the opposite (the higher survival of *C. septempunctata*). Claiming any apparent advantage of *H. axyridis* over *C. septempunctata* in exposure to thiacloprid would require a carefully designed field study in order to confirm the laboratory results.

On the other hand, our study may suggest that in the case of thiacloprid, both ladybirds are sensitive to the dose rate of the product used against their prey, and their survival is higher at the lower dose, for most of the time. This last conclusion is nevertheless vulnerable to criticism, as in our study the data on thiacloprid seem too inconsistent for valid conclusions.

## 5. Conclusions

1. Of the two tested insecticides, lambda-cyhalothrin kills most instars of *Coccinella septempunctata* and *Harmonia axyridis* within a 3 h period since the start of ingestion of intoxicated prey. The action of thiacloprid to which the predators are exposed by the same route is more extended in time in both the ladybirds, and more variable between their instars. It also allows for the survival of a considerable number of individuals beyond 48 h.
2. The applications of lambda-cyhalothrin against aphids are likely to impair population dynamics to the similar extent in *C. septempunctata* and *H. axyridis*, whereas the applications of thiacloprid cause different response in the two predator species. The most apparent difference between the species was found in L4 larvae, where higher survival of the L4 instar was observed in *H. axyridis* compared to *C. septempunctata*, irrespective of the dose rate used.
3. Reducing the field rate of the insecticides containing lambda-cyhalothrin has no practical value for the survival of either of the coccinellid species tested. Contrastingly, while applying thiacloprid insecticides, using half of the full field rate of the product instead may probably enhance the chances of survival in L1–L2 and, to a lesser degree, in L4 larvae, of both ladybird species.

## Figures and Tables

**Table 1:** Significance of differences between survival dynamics of *Harmonia axyridis* and *Coccinella septempunctata* after their foraging on insecticide-treated prey.

Product Code; Percentage of the Recommended Field Rate [%]	Instar *	Z **	*p*
KARATE; 100	L1	−0.514	0.610
	L2	0.000	1.000
	L3	−2.044	0.040
	L4	−2.563	0.010
	A	−2.044	0.040
KARATE; 50	L1	−1.540	0.12
	L2	−0.566	0.570
	L3	−0.559	0.580
	L4	−0.228	0.820
	A	−0.960	0.340
CALYPSO; 100	L1	−1.633	0.100
	L2	−2.819	0.005
	L3	4.036	0.005
	L4	4.004	0.005
	A	1.217	0.220
CALYPSO; 50	L1	−0.278	0.781
	L2	−1.950	0.051
	L3	−2.413	0.016
	L4	3.910	0.000
	A	0.041	0.968

* A—adults, L1, L2, L3, L4—larval stages. ** Statistical significance is shown for pairwise comparisons of Kaplan-Meier survival functions for *H. axyridis* vs. *C. septempunctata*, for adults and larval instars separately, in each (product × dose rate) treatment. Survival dynamics are assumed to be significantly different at *p* ≤ 0.05. The survival functions compared are plotted pairwise in the Figure 1, Figure 2, Figure 3 and Figure 4.

## Data Availability

Data are available upon request from the authors.
